# Evolutionary shifts in condition-transfer effects but not anticipatory maternal effects in Trinidadian killifish (*Anablepsoides hartii*)

**DOI:** 10.1098/rspb.2026.1362

**Published:** 2026-11-04

**Authors:** Marcus A. Lee, Amanda J. Stoner, Christopher Roden, Matthew R. Walsh

**Affiliations:** ^1^ Department of Biology, Lund University, Lund, Skåne County 223 62, Sweden; ^2^ Department of Biology, The University of Texas at Arlington, Arlington, TX 76019, USA; ^3^ Department of Biology, University of Miami, Coral Gables, FL 33124, USA

**Keywords:** maternal effects, life-history evolution, predation risk, resource limitation

## Abstract

Transgenerational plasticity (TGP) shapes offspring phenotypes independent of changes in DNA sequence. However, it remains unclear whether transgenerational plasticity primarily reflects adaptive anticipatory adjustments to predicted future environments or condition-dependent carryover from the parental energetic state. Because traits differ in their functional roles within life-history architecture, selection may favour distinct cross-generational mechanisms depending on the ecological context. We tested these alternatives using Trinidadian killifish (*Anablepsoides hartii*), which are locally adapted to either high-predation (HP) or predator-free killifish-only (KO) environments. Predation regime structures population density and per capita resource availability, making food availability an ecologically realistic proxy for the pathways through which predation regimes influence maternal conditions and transgenerational responses. Therefore, we experimentally manipulated the maternal (F2) and offspring (F3) food environments using a split-brood design and quantified the offspring body mass, body condition and egg development rate. Offspring body mass was overwhelmingly determined by the immediate food environment. By contrast, the egg development rate was strongly influenced by the maternal food environment. This condition-transfer effect was much stronger in the high-predation population. We found little evidence of anticipatory effects for any trait. Our results indicate that the evolution of condition-dependent maternal effects may represent a primary adaptive pathway when selection acts strongly on life-history pacing, helping reconcile mixed empirical support for anticipatory transgenerational plasticity.

## 1. Introduction

Environmental conditions often fluctuate across generations, requiring organisms to contend with not only their immediate surroundings but also with the ecological legacies inherited from their parents. Phenotypic plasticity allows organisms to respond to their current environment within a generation, whereas transgenerational plasticity (TGP) provides a pathway through which parental environments shape offspring phenotypes independent of changes in DNA sequence. Such transgenerational effects can arise through altered provisioning [1,2], physiological state [3] or epigenetic mechanisms [4], and are increasingly recognized as important components of life-history evolution [5–8]. However, a central question remains: do transgenerational effects represent adaptive anticipatory adjustments to future environments or do they primarily reflect condition-dependent carryover from parental experiences [9–12]?

Interest in TGP or maternal effects has grown rapidly across diverse taxa—from microbes and fungi to plants and animals—reflecting its potential importance in ecology and evolution [13–20]. However, TGP is a broad term that encompasses multiple mechanistic pathways [21]. Anticipatory effects refer to parental modifications that function as preparatory adjustments, enabling offspring to better match predicted future environments when ecological conditions are reliably transmitted across generations [12]. While there are clear examples of such ‘anticipatory maternal effects’ [1,13,16,18,20,22–28], the generality of this maternal effect remains controversial. This is because many organisms experience environmental conditions that theory clearly predicts should favour the evolution of anticipatory maternal effects [27,29–33]. However, an influential meta-analysis described the current empirical evidence for ‘anticipatory maternal effects’ as ‘weak’ [12]. Alternatively, condition-transfer posits that the offspring phenotype may simply reflect a carryover, whereby the parental energetic state or stress physiology passively shapes developmental trajectories [34–38]. Because both the mechanisms can generate cross-generational resemblance, distinguishing predictive effects from condition-transfer remains a central challenge in understanding the evolutionary significance of TGP [9,10].

One factor that may contribute to this ambiguity is the functional role of the focal trait. Indeed, maternal effects do not act uniformly but vary in strength among traits with distinct functions [39]. This may be partly owing to traits differing in the degree to which their fitness consequences depend on matching specific environmental states versus responding flexibly to immediate conditions. For example, discrete morphological traits that are expressed in response to predictable predator presence may be more likely to evolve anticipatory TGP [40]. Conversely, the life-history traits that are tightly coupled to parental energetic state, such as offspring growth or number [41], may be more strongly influenced by condition-transfer effects. As energy budgets constrain both defence production and reproductive allocation, parental condition may mediate offspring phenotype regardless of whether environmental matching is favoured by selection. As a result, energetic trade-offs may blur the boundary between anticipatory and condition-transfer mechanisms because the parental state reflects both ecological conditions and constrains the expression of costly traits.

Predation is perhaps one of the most pervasive selective forces that shape life-history evolution. Predators increase extrinsic mortality and are commonly associated with additional ecological shifts that can modify selection on prey (i.e. lower densities, higher resources, etc.). As a result, predation often favours the coordinated evolution of earlier maturation, more offspring and shifts in energy allocation towards reproduction at the expense of growth [42–46]. By contrast, where predation is relaxed, populations typically evolve slower life histories [47], investing in competitive advantages such as greater size [48] and more aggressive behaviours [49,50]. Therefore, predation acts both directly and indirectly on the evolution of species and alters the energetic context in which parental effects operate. Although many species have documented predator-induced responses that extend beyond a single generation [13,51,52], it remains unclear whether contrasting predation regimes favour the evolution of anticipatory plasticity or condition-transfer effects. If predator presence is predictable across generations, selection could favour anticipatory adjustments that prepare offspring for high-risk environments. Alternatively, if rapid developmental timing is critical for fitness under high mortality, a tighter coupling between maternal condition and offspring traits may evolve.

Trinidadian killifish (*Anablepsoides hartii*) provide a powerful natural system to address how contrasting ecological conditions may select for alternative TGP mechanisms. Unlike many annual killifish, *A. hartii* is a relatively long-lived species, with individuals capable of surviving for multiple years in the wild, allowing parents to experience substantial variations in environmental conditions over their lifetime. Along the Northern mountain ridge, multiple streams across separate drainage basins exhibit similar morphologies. More specifically, waterfalls create barriers that inhibit the upstream migration of large predatory fish and guppies (*Poecilia reticulata*), meaning that *A. hartii* are located in high-predation (HP) sites with numerous large predators versus killifish-only (KO) sites where they are the only fish present [53,54]. This consistent divergence in predation level is strongly associated with shifts in life-history evolution, including traits such as reproductive maturation [46,55]. Fish from HP populations have reduced effective population sizes and, in turn, experience an increase in per capita resources [56,57]. By contrast, KO populations exhibit high population density and greater competition for resources. Although the majority of abiotic factors are similar [54], there are some ecological covariates, such as canopy openness, that interact with seasonal dynamics to shape both the differential abundance and the predictability of resources at these sites. For example, a substantial dietary component for *A. hartii* is terrestrial invertebrates and open HP sites tend to receive relatively consistent inputs of terrestrial invertebrates, whereas KO populations are more strongly structured by seasonal fluctuations in resource availability [58]. This divergence in ecological conditions—and critically, in the variation of those conditions—has the potential to generate differential selective pressures that promote the evolution of alternative TGP mechanisms.

To test whether populations that have evolved under contrasting ecological conditions differ in the mechanisms underlying TGP, we compared the relative importance of condition-transfer and anticipatory maternal effects in HP and KO populations. Second-generation laboratory-born fish from three replicate streams were reared under contrasting food levels designed to mimic the differences in growth observed between the HP and KO environments. Using a split-brood design, we experimentally manipulated food availability in the third laboratory generation to quantify the relative contributions of maternal and current environmental conditions to offspring life-history traits. We focused on traits that occupy distinct positions within the life-history architecture of this system: offspring body mass, body condition and egg development rates. Body mass and condition reflect resource acquisition and allocation during development and are therefore expected to respond strongly to immediate environmental conditions, with a larger size potentially enhancing early competitive ability and faster allocation reducing the time until reproductive maturity under predation risk. By contrast, egg development rate governs the timing of offspring entry into the environment and may be particularly sensitive to selection on reproductive pacing, where faster development can reduce exposure to pre-hatching mortality and accelerate recruitment under high-risk conditions. If transgenerational effects operate primarily through anticipatory mechanisms, we would expect post-hatching offspring traits to reflect matching between maternal and offspring environments, particularly in populations experiencing stable predation regimes (i.e. fish from HP sites; figure 1). Alternatively, if condition-transfer dominates, maternal energetic state should directly influence egg development, irrespective of environmental matching, with stronger coupling expected in populations where rapid life-history pacing enhances fitness (i.e. high predation sites). By comparing populations that differ in predation history, this system allows for the evaluation of whether ecological divergence modifies the strength or form of cross-generational plasticity. We show here that predation history does indeed strengthen condition-transfer effects on egg development rate but provides little evidence for anticipatory maternal effects across traits.

**Figure 1.**
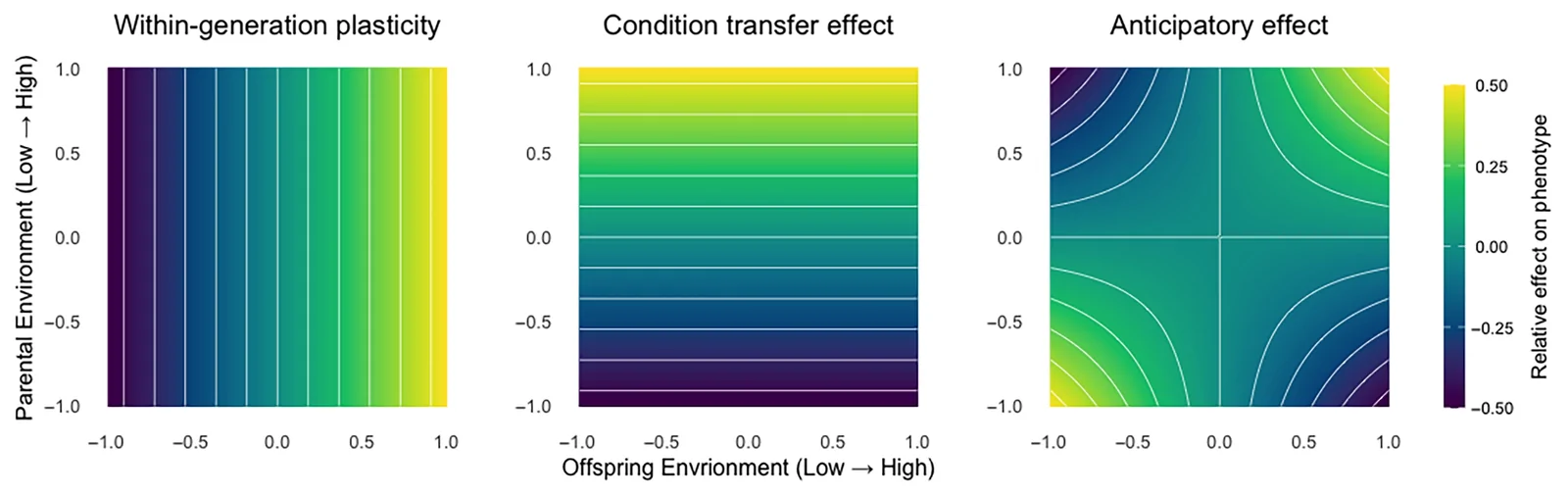
Three hypothetical phenotypic landscapes determined by differing evolutionary mechanisms for maintaining fitness across ecological gradients. Within-generation plasticity is entirely driven by the offspring environment, whereas condition-transfer effects are entirely correlated with the parental environment. The adaptive anticipatory effects in this example are derived from the match-mismatched hypothesis, leading to the greatest contribution along the positive diagonal axis. The lighter the colour scale, the stronger is the effect.

## 2. Materials and methods

### (a) Fish collection and rearing

Wild F0 killifish were sampled from HP and KO locations from three south-facing rivers of the Northern Range mountains of Trinidad and Tobago in June 2023; the Arima (AM—10°41′12.552′′N 61°17′45.7434′′W), Aripo (AP—10°40′34.4634′′N 61°15′41.2554′′W) and El Cedro (EC—10°39′50.148′′N 61°15′48.456′′W). These rivers were selected because their close geographic proximity minimizes broad-scale differences in ecological conditions (such as temperature, conductivity, pH and oxygen) [54,55], while their occurrence in separate drainage basins provides independent evolutionary replicates.

Approximately 30 individuals were collected from each site using dip nets. They were then transported to the laboratory facilities at The University of Texas at Arlington in late June 2023, where the fish were maintained at 25 ± 1°C on a 12 h light : 12 h dark photoperiod. Each female was given an individual ID and housed individually in a 9 l aquarium tank with a single male (also with an individual ID) from the same locality and artificial spawning substrate. Eggs were removed daily from the spawning substrate and placed in a single well of a 24-well plate. Each well contained water and methylene blue solution at a concentration of 4 ppm, to prevent fungal growth. After hatching, the F1 larvae were relocated to 1 l aquaria and reared on an ad libitum diet of brine shrimp nauplii and commercial flake food. During growth, fish were periodically rehoused in larger aquaria to provide size-appropriate tanks up to a maximum volume of 9 l. All fish rearing and subsequent experiments were performed in the same laboratory. All aquaria were haphazardly repositioned within the temperature-controlled room at regular intervals to minimize any position-specific environmental differences.

At the point of reproductive maturity, determined by the appearance of ‘bars’ along the top and bottom of the caudal fin in males, a single female and male were paired together in a 9 l aquarium with an artificial spawning substrate. These pairings were matched within the same locality and the F2 generation was produced. All pairings were constructed to ensure no full-sibling mating (see supplementary material figure S1 and table S1 for final pedigree sample sizes). Thus, genetic diversity from the original wild-caught fish was maintained. The F2 generation of lab-born fish was hatched from eggs between March and October 2024. Upon hatching, larvae were reared individually in 0.5 l aquaria. All fish were immediately assigned to one of two feeding regimes: (i) high food (HF)—provided ad libitum brine shrimp nauplii or (ii) low food (LF), where the fish received restricted rations of brine shrimp designed to mimic growth at KO sites (see [46]). In order to accommodate growth, the LF ration was increased every 2 weeks from the date of hatching (from 5 to 140 µl of brine shrimp nauplii). All fish received the same ration for each 2 week period, regardless of differences in size. These increments occurred 18 times which represents approximately 8 months. Given that resource levels can alter developmental timing, this feeding regime was constructed to exceed the time until reproductive maturity, even at LF levels (see [46]). Mature F2 individuals were then paired to produce the F3 generation, following the same procedure as prior generations, i.e. within population, no inbreeding and size of the mating aquarium. F3 offspring were allocated to either HF or LF conditions using a split-brood design. Every effort was made to produce an equal sample size for each treatment subgroup (supplementary material, table S2). However, various factors precluded a balanced design, such as unproductive pairings, infertile eggs and early life mortality, all of which were more prominent under LF conditions.

### (b) Data collection and analysis

We quantified female body condition, egg development rate and offspring (F3) size and body condition after 30 days of growth. F2 mothers were weighed and measured at the time of pairing with a male using an analytical balance accurate to 0.1 mg (VWR-14AC, VWR International, Randor, PA, USA) and digital callipers accurate to 0.01 mm (Thermo Fisher Scientific, Waltham, MA, USA). Owing to the tight theoretical coupling of maternal, not paternal, condition indices on the next generation, only female F2 individuals were measured [59]. Each egg laid was photographed within 24 h and measured using ImageJ [60]. Egg development time was calculated from the date laid to the date of hatching. F3 individuals were measured 30 days post-hatching, as the F2 generation. Body condition was calculated for both the F3 individuals and F2 mothers according to Fulton's condition factor (K-Factor), which is K = (weight × 100) / length^3^ [61]. Equivalent data were not collected until the maternal effects associated with wild collection were removed through subsequent generations of laboratory rearing. Consequently, comparable estimates of weight, body condition and development rate are not available for founding individuals or the F1 generation. The data and associated codes are available in the Dryad repository [62].

All analyses were performed and figures were produced using R v.4.4.2 [63]. Weight and condition analyses were conducted using Bayesian animal models implemented in the brms package [64,65]. Specifically, models were fitted using Hamiltonian Markov chain Monte Carlo sampling with 6000 iterations and 2000 burn-ins across four chains. The response variable (F3 body weight or F3 body condition) was modelled with a log-normal likelihood, with the expected phenotype determined by F2 food level, F3 food level, population and their interactions. Preliminary analyses, including river identity, yielded parameter estimates that were qualitatively unchanged from those of the simpler model (supplementary material, table S3). Therefore, the river was excluded from the final Bayesian analyses in favour of the more parsimonious model. Residual variation was partitioned into additive genetic variance structured by the pedigree-relatedness matrix (A) and environmental variance. Weakly informative priors constrained implausible effect sizes while remaining non-restrictive. These priors were evaluated with a sensitivity analysis using both weaker and stronger priors and were found not to meaningfully influence posterior estimates. Parameter uncertainty was reported as 95% credible intervals from the posterior distribution. For egg development, a number of observations were recorded without sufficient date resolution or conflicting time scales, either as the egg laid date or the hatch date. In an attempt to be conservative, all such questionable dates were treated as not available (NA), which prevented the ability to determine the development time for any individual with at least one NA. Consequently, the additive genetic variance component was not identifiable and the Bayesian model showed persistent non-convergence across priors and sampler settings. Therefore, egg development was analysed using a generalized linear model with a Poisson distribution. Because additive genetic effects could not be estimated in the reduced egg development dataset, river identity was included as an additional fixed effect to account for potential baseline differences among rivers. This model was subjected to backwards selection based on the ΔAIC (Akaike Information Criterion) values. To further explore any correlations between egg development and maternal condition, egg diameter or population, we performed several generalized multiple regression models with Poisson distribution.

For interpretive clarity, detecting significant effects between F3 food levels denotes the current environmental effects (within-generation plasticity); whereas differences between F2 food levels indicate the presence of condition-transfer effects (maternal effects). The detection of an interaction between the two is a prerequisite for anticipatory effects, but also depends on the direction to indicate adaptive potential. Any differences, including population, would suggest evolved differences between the HP and KO sites.

## 3. Results

### (a) Offspring size

F3 body weight was strongly influenced by the food environment experienced by the offspring (figure 2). Individuals raised under HF conditions were substantially heavier than those raised under LF conditions (*β* = 0.61, 95% CI = 0.58–0.65; figure 3A), indicating that the immediate developmental environment was the dominant determinant of body mass. The maternal food environment had a much smaller, yet still positive effect, such that offspring whose mothers experienced HF were slightly heavier on average than those whose mothers experienced LF (*β* = 0.04, 95% CI = 0.01–0.08; figure 2). There was little evidence of an overall difference in body weight between populations (*β* = 0.01, 95% CI = −0.03 to 0.05). However, the response to offspring food differed slightly between populations (F3 food × population: *β* = −0.03, 95% CI = −0.06 to 0.00), suggesting that the increase in body weight under HF conditions was marginally weaker in the KO population (figure 2). The interaction between maternal and offspring food environments was similarly small (F3 food × F2 food: *β* = −0.03, 95% CI = −0.06 to 0.00), providing only weak evidence that the maternal environment modified the offspring response to current food conditions. Other interaction terms were centred near zero, with credible intervals overlapping zero. Overall, the model explained approximately 24% of the variation in body weight (Bayesian *R²* = 0.24, 95% CI = 0.21–0.26). The animal random effect indicated moderate among-individual variation associated with relatedness (s.d. = 0.10, 95% CI = 0.02–0.17), corresponding to a narrow-sense heritability of approximately 0.06 (95% CI = 0.003–0.16).

**Figure 2.**
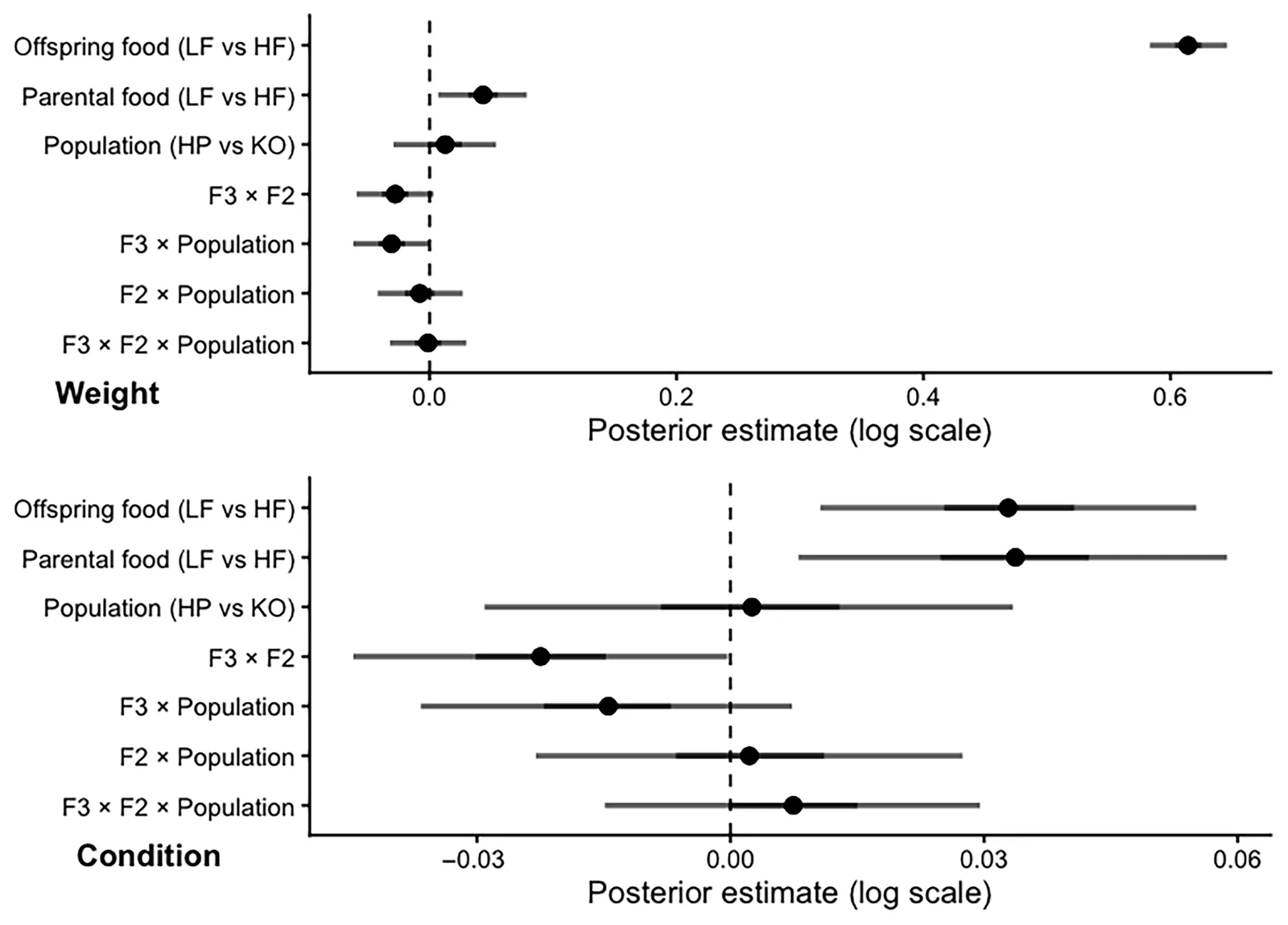
Forest plot of the fixed effect posterior estimates derived from each of the Bayesian mixed models. The points represent the median, with the dark bars denoting the 50% confidence interval (CI) and the grey bars denoting 95%. The dashed vertical line represents zero, i.e. no effect. Estimates whose credible intervals do not overlap with zero indicate stronger support for directional effects, whereas overlapping intervals indicate greater uncertainty.

**Figure 3.**
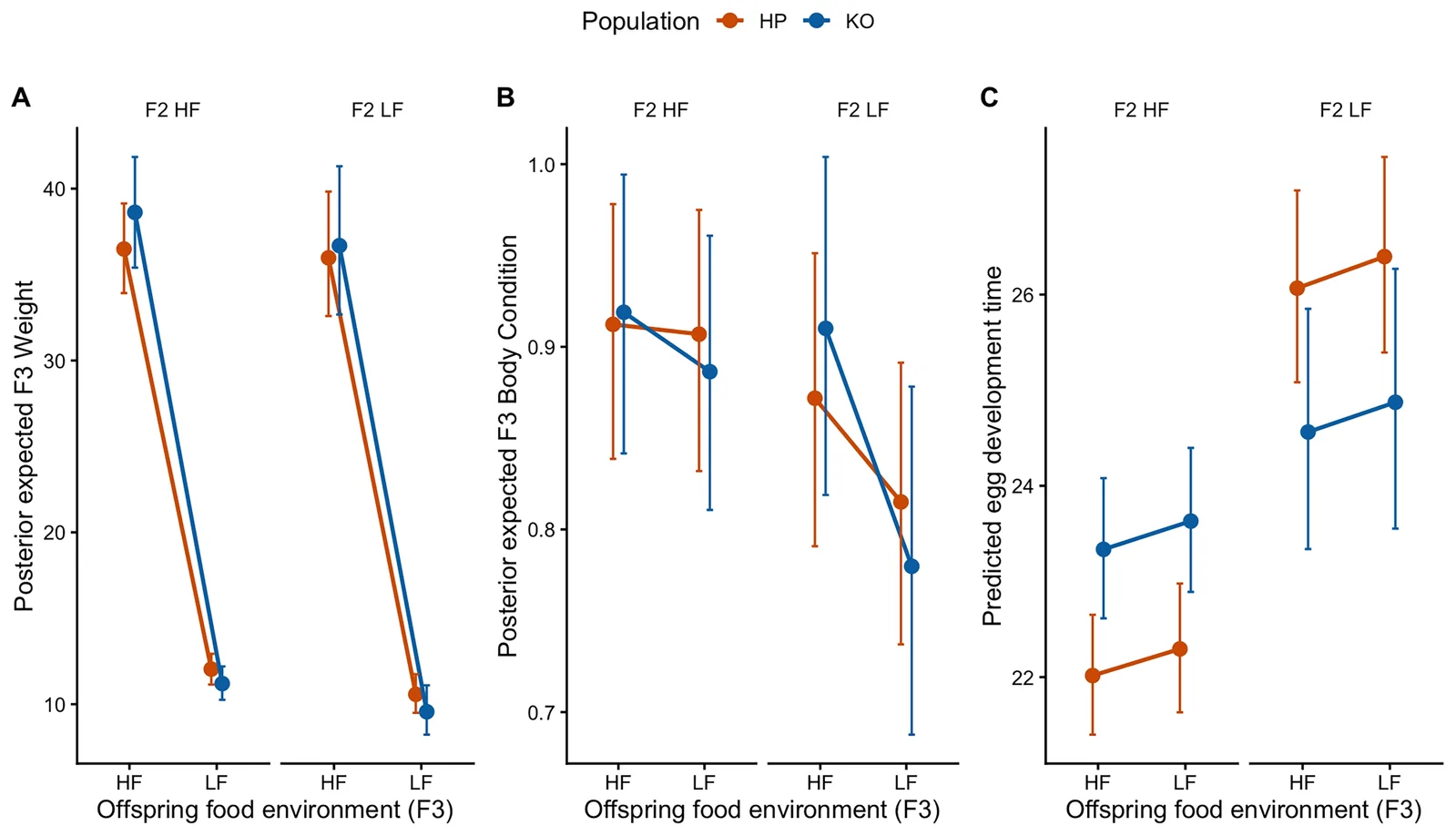
Reaction norms for the three dependent variables: (A) F3 body weight (mg), (B) F3 body condition (K) and (C) egg development rate (days). Each panel is subdivided by maternal food treatment in the F2 generation (high food, HF; low food, LF). Panels A and B represent the posterior predicted mean from Bayesian mixed models, whereas panel C represents the back-transformed predicted mean from a Poisson generalized linear model. Error bars denote the 95% credible interval in panels A and B and 95% confidence interval (CI) in panel C.

### (b) Offspring body condition

The body condition of the F3 generation showed only weak responses to environmental treatments (figure 2). Offspring raised under HF conditions had slightly higher body condition than those raised under LF conditions (*β* = 0.033, 95% CI = 0.011–0.055; figure 3B) and a similarly small positive effect of maternal food environment was detected (*β* = 0.034, 95% CI = 0.008–0.059), indicating that both offspring and maternal environments contributed modestly to the variation in body condition. There was little evidence of population differences (*β* = 0.002, 95% CI = −0.029 to 0.033). The interaction between offspring and maternal food environments was small but slightly negative (*β* = −0.022, 95% CI = −0.045 to −0.0003), suggesting that the combined effect of LF across generations was marginally stronger than expected from their independent effects (figures 2 and 3B). Other interaction terms were centred near zero and their credible intervals broadly overlapped with zero. Overall, the model explained very little variation in body condition (Bayesian *R²* = 0.014, 95% CI = 0.005–0.027), indicating that most individual variation in body condition occurred independently of the experimental treatment. The animal random effect also indicated a low among-individual variance associated with relatedness (s.d. = 0.09, 95% CI = 0.04–0.14), corresponding to a very low estimated narrow-sense heritability for body condition (median *h^2^* = 0.009, 95% CI = 0.00002–0.062).

### (c) Egg development rate

Egg development time varied strongly by the river of origin (χ2 = 197.155, d.f. = 2, *p* <0.001, table 1), indicating local adaptation within the drainage basins (supplementary material, figure 2). Experimentally, we primarily detected variations in the maternal food environment rather than the offspring food environment (figure 3C). In the reduced model, eggs produced by mothers experiencing LF developed slightly slower than those from mothers experiencing HF (*β* = 0.169 ± 0.022 s.e., *z* = −7.66, *p* < 0.001), corresponding to a modest increase in development time of approximately 3 days under unfavourable maternal conditions. By contrast, the offspring food environment had only a weak and non-significant effect on the development time (*β* = 0.013 ± 0.016 s.e., *p* = 0.42). Baseline development time differed between populations (*β* = 0.058 ± 0.019 s.e., *z* = 3.08, *p* = 0.002), but the maternal food effect also varied between populations (F2 food × population: *β* = −0.118 ± 0.036 s.e., *z* = −3.266, *p* = 0.001), indicating that the influence of maternal food on the development rate differed across populations. Specifically, the HP populations were far more sensitive to variations in the F2 feeding regime than the KO population (figure 3C). The full factorial model used for conceptual comparisons produced similar results: maternal food remained the dominant predictor of egg development time, whereas interactions involving offspring food were weak and generally unsupported. Model comparison supported the reduced model (AIC = 4031) over the full interaction model (AIC = 4034).

**Table 1.** Egg development rate model results.

term	*β*	s.e.	*z*	95% CI	*p*-value
intercept	26.1	0.019	170	25.1–27.1	
F3 food	1.01	0.016	0.80	0.98–1.04	0.42
F2 food	1.18	0.022	7.66	1.13–1.24	<0.001
population	1.06	0.019	3.08	1.02–1.10	0.002
Aripo river	0.77	0.019	–13.7	0.74–0.80	<0.001
El Cedro river	0.78	0.022	–11.0	0.75–0.82	<0.001
F3 × F2	0.99	0.035	–0.26	0.93–1.06	0.80
F2 × population	0.89	0.036	–3.27	0.83–0.95	0.001
F3 × population	1.01	0.032	0.46	0.83–0.96	0.65
F3 × F2 × population	0.88	0.075	–1.67	0.76–1.02	0.1

To explore the potential underlying mechanisms of maternal effects on egg development time, we examined the relationships among maternal condition, egg size and development rate. Overall, egg diameter was not associated with development time (*β* = –0.094 ± 0.071 s.e., *z* = –1.321, *p* = 0.19; figure 4A). This relationship differed between populations (egg diameter × population: *β* = 0.21 ± 0.111 s.e., *z* = 1.9, *p* = 0.057), with KO populations exhibiting a positive relationship and HP populations a negative relationship. There was no significant relationship between maternal body size and egg size (*β* = 0.002 ± 0.002 s.e., *p* = 0.22). Maternal body condition, on the other hand, was strongly associated with egg development time (*β* = −0.742 ± 0.175 s.e., *z* = −4.25, *p* < 0.001), with offspring from mothers in better condition developing substantially faster (figure 4B). This effect also differed between populations (maternal condition × population: *β* = 0.505 ± 0.234 s.e., z = 2.161, *p* < 0.001), with the HP population showing far more susceptibility to F2 body condition than the KO population. Despite this, egg diameter showed little relationship with maternal body condition (*β* = 0.002 ± 0.002 s.e., *z* = 1.22, *p* = 0.22) nor the interaction between maternal condition and population (*β* = 0.004 ± 0.002 s.e., *z* = –1.53, *p* = 0.13; figure 4C). Together, these results suggest that the observed maternal effects on egg development time are unlikely to arise primarily through variation in egg size but instead reflect condition-dependent differences in egg quality or developmental physiology.

**Figure 4.**
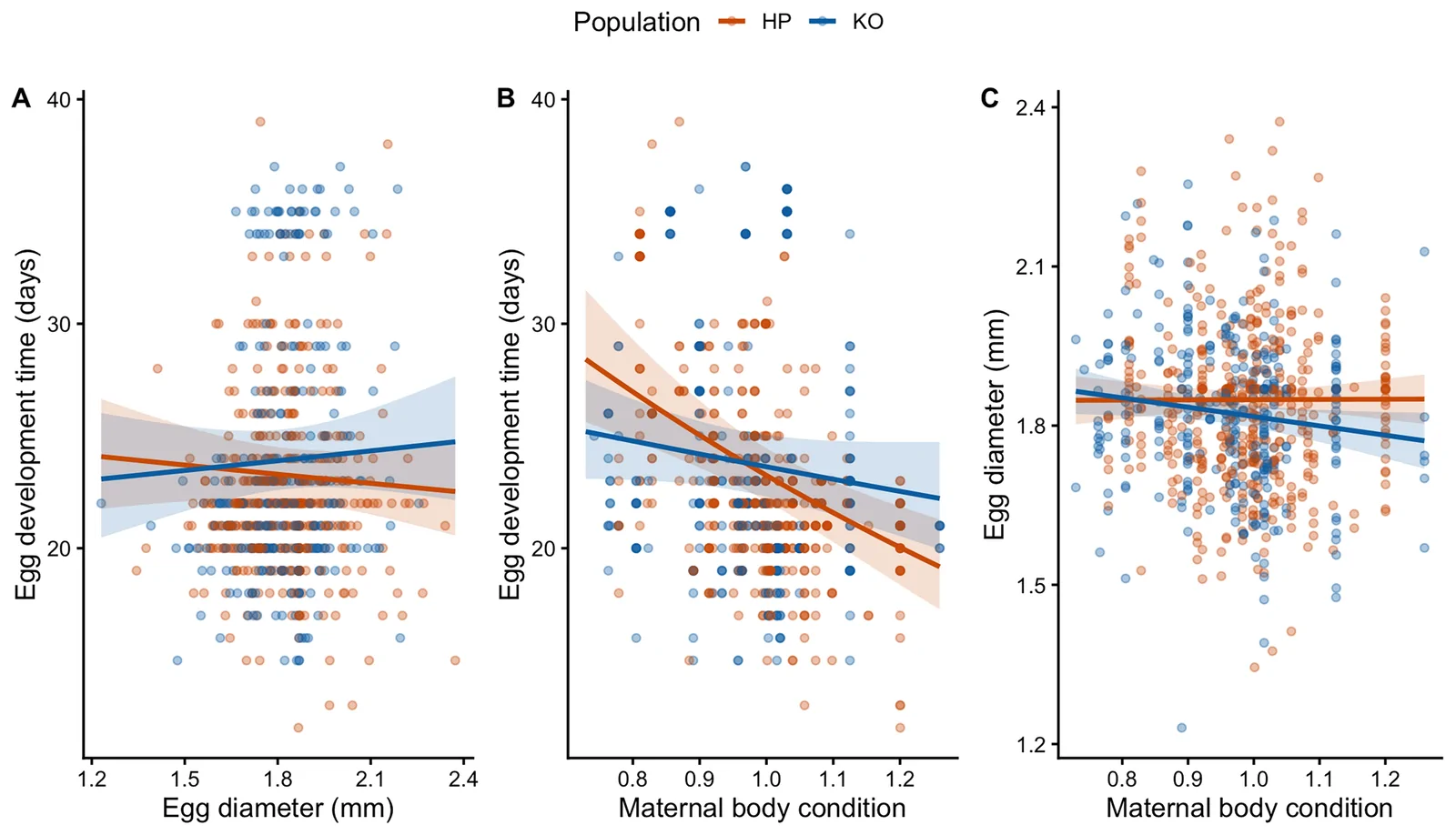
Relationships among maternal (F2) body condition, egg size and egg development time: (A) egg development as a function of egg diameter, (B) egg development as a function of maternal condition and (C) egg diameter as a function of maternal condition. The points represent the raw data, coloured by population (HP and KO). The lines represent the model-predicted means and the shaded ribbons indicate 95% confidence intervals (CI). Panels A and B show predictions from Poisson generalized linear models, whereas panel C shows predictions from a Gaussian linear model.

## 4. Discussion

Our results clearly demonstrate that the strength of within- versus between-generation plasticity mechanisms is trait dependent. More specifically, our results indicate that transgenerational effects primarily exert influence through condition-transfer effects, as opposed to anticipatory matching between offspring and parental environments in this system. At the trait level, within-generation plasticity was the strongest determinant of offspring weight, whereas the egg development rate was determined by the maternal environment. Body condition demonstrated an interaction between the two environments, but inspection revealed a compounding negative effect of repeated resource-depauperate environments. Although heritability estimates were low, particularly for body condition, they were non-zero, indicating the capacity for an evolutionary response. However, the dominance of the environmental and maternal variance components suggests that plastic pathways play a larger role in shaping phenotypic expression. This pattern aligns with condition-transfer effects and not predicative provisioning of future environments, reflecting that in this system, the primary mechanism determining the evolution of maternal effects is resource meditated rather than information mediated.

Although the detected maternal effects were largely consistent with condition-transfer, their magnitude differed between predation regimes (figure 5), suggesting that evolutionary history has modified how maternal nutritional state influences offspring phenotype. Maternal food availability had comparatively weaker effects on offspring growth traits in HP populations than in KO populations, whereas its influence on egg development time was greater in HP populations than in the KO populations. These contrasting patterns indicate that the strength of maternal effects has evolved differently for different traits. Despite these differences in magnitude, the observed patterns remained consistent with condition-transfer rather than anticipatory maternal effects being present across ecological contexts.

**Figure 5.**
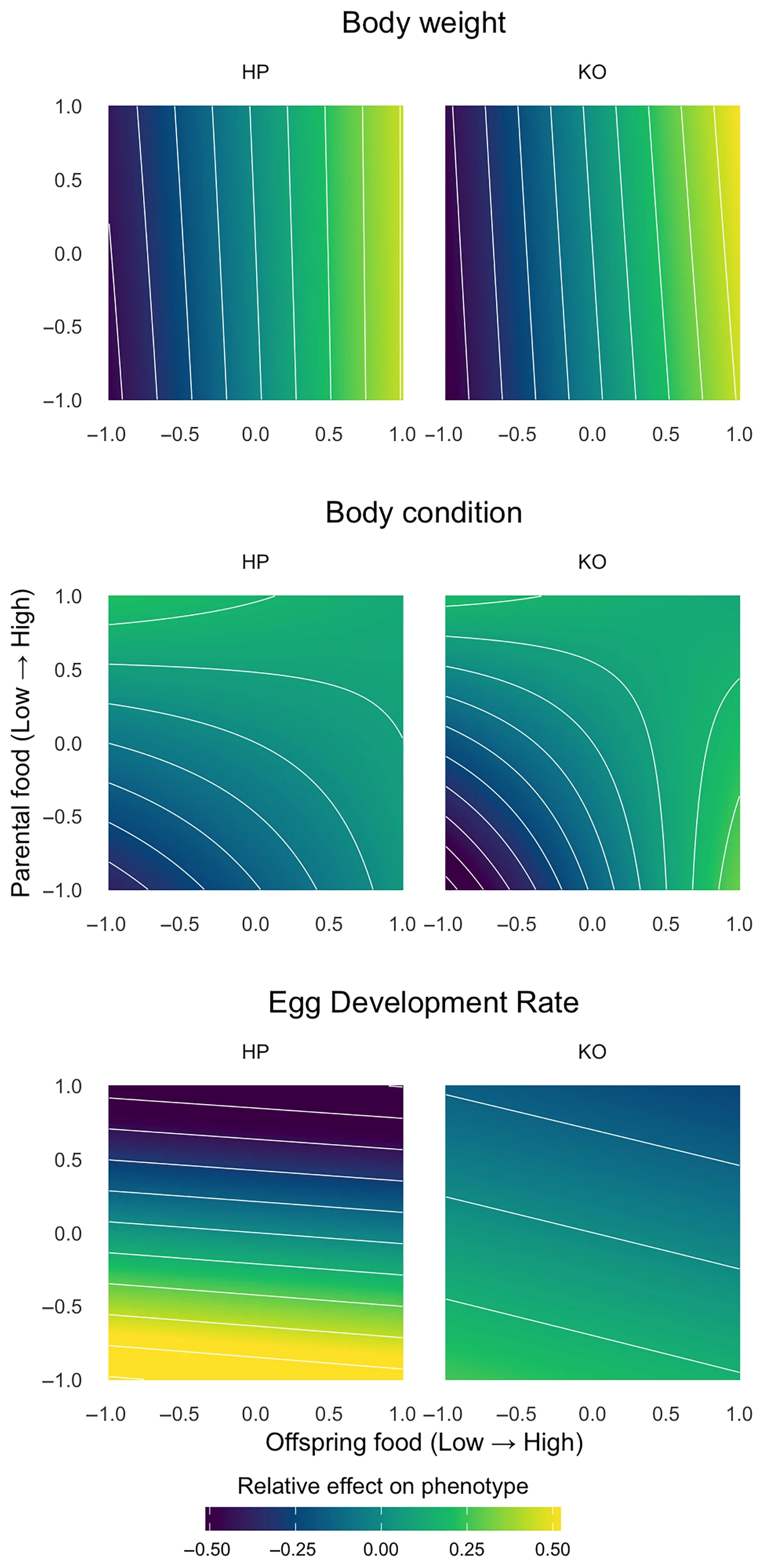
Visualization of the phenotypic responses according to the relative influence of within-generation plasticity, condition-transfer effects and anticipatory effects extracted as estimates from related models. Within-generation plasticity produces a gradient along the offspring environment axis, condition-transfer produces a gradient along the parental environment axis, and anticipatory effects produce a ridge or valley where offspring and parental environments match or mismatch. The colour scale shows the relative effect on the phenotype, with positive and negative values indicating the direction of the standardized response across environmental gradients. Each row represents a different trait, with the left column denoting HP populations and the right column denoting the KO populations.

Variation in predation regimes provides powerful selective forces that directly and indirectly shape life-history strategies, often favouring earlier reproduction, altered growth trajectories and shifts in energy allocation [43,55,57]. These life-history trade-offs have been shown to exist in our focal species at the sample sites, with those experiencing HP maturing earlier, producing fewer offspring and behaving less aggressively [46,50,55]. Encouragingly, we found very consistent results surrounding egg size and development rate in multiple common garden experiments, from over two decades [46] to more recently [66]. This firmly suggests that HP populations have evolved to exhibit rapid reproduction and development. Furthermore, we observed a stronger negative correlation between maternal condition and egg development rate in these populations (figure 4B). Exposed to high-resource environments, such as HP sites, mothers in better condition produce faster-developing eggs. Under such conditions, *A. hartii* are often confined to marginal habitats where predation risk is reduced; however, this increases the risk of egg desiccation owing to frequent fluctuations in stream depth [66]. Therefore, early hatching probably enhances offspring survival in risky environments despite the potential increase in exposure to predation [67]. Taken together, such a pattern suggests that the multifaceted nature of selection in HP environments strengthen the linkage between maternal condition and offspring developmental rate, potentially amplifying condition-dependent maternal effects.

As mentioned previously, our results demonstrate a decoupling of mechanisms governing growth and developmental timing. Offspring mass was overwhelmingly determined by the immediate resource level, whereas the egg development rate was primarily shaped by the maternal resource level. Strikingly, maternal influence on development did not appear to operate through variations in egg size (figure 4A). Although larger eggs required marginally longer to develop in KO populations (figure 4A), the egg diameter was largely independent of maternal condition for HP populations and thus cannot explain the accelerated development observed from HF mothers. Instead, these findings suggest that maternal effects influence developmental tempo through changes in egg quality rather than egg quantity. Such changes could arise through altered provisioning of nutrients or other bioactive compounds, including hormones and maternally deposited regulatory molecules [68,69]. During early embryogenesis, development is directed largely by maternally supplied transcripts before the activation of the embryonic genome [70] and variation in the abundance or composition of these mRNAs and small regulatory RNAs could therefore modify developmental trajectories without requiring changes in egg size [71]. Similarly, epigenetic modifications, including DNA methylation and histone modifications, may alter the patterns of embryonic gene expression later in development. While our data cannot distinguish among these mechanisms, they demonstrate that maternal nutritional history can influence the developmental rate independently of structural investment per offspring [72], highlighting the need for future work linking maternal provisioning with the molecular regulation of embryogenesis.

This trait-specific architecture has broader implications for understanding when condition-transfer versus anticipatory effects is expected to evolve [8,73]. The development rate governs when offspring enter the environment and begin resource acquisition; therefore, they are particularly sensitive to selection on reproductive timing [74]. By contrast, body mass can remain plastic and responsive to the changing conditions. This suggests that maternal effects may be more tightly integrated with life-history pacing than with offspring size *per se*. If so, this perspective highlights a particular challenge with the match-mismatch conceptualization in the evolution of anticipatory effects, which require reliable environmental predictability across generations and selection for offspring phenotypes that match future conditions [8,11,20]. When the key fitness axis is timing rather than phenotype matching *per se*, strong anticipatory forecasting may simply be unnecessary. The maternal energetic state already encapsulates ecologically relevant information about the current environment. If environment shifts between the time the egg is laid and the offspring hatching, within-generation plasticity can make accommodations to maintain fitness. Under this scenario, selection may favour a tighter coupling between the maternal state and developmental tempo rather than the evolution of predictive adjustments. Therefore, our results suggest that condition-transfer effects may not just represent a constrained or non-adaptive alternative to anticipatory effects. Instead, they may represent the primary adaptive pathway in systems in which life-history pacing is the dominant selective target. Despite finding no evidence for anticipatory effects, we suggest, based on the above logic, that their evolution may be more restricted to systems where selection on offspring phenotype is more tightly linked to specific and predictable dichotomies (e.g. predator- or seasonal-morphs) [75–77].

One consideration worth mentioning is the potential for the ancestral ecological context to influence the interpretation of the evolutionary trajectory. Because upstream predator-free KO populations are thought to arise via colonization from downstream HP environments [46] and have molecular signals of shared ancestry [78], HP fish could be considered the ancestral ecological state in *A. hartii*. In this light, the heightened sensitivity of the developmental rate to maternal conditions observed in HP populations may reflect the retention of ancestral maternal effects, and the reduced sensitivity observed in KO populations may be a relaxation of selection on rapid developmental pacing following predator release. Logically, the fitness advantage of accelerating development via maternal conditions may weaken in the absence of predators, allowing the strength of condition-dependent maternal effects to attenuate. Although resolving ancestral states lies beyond the scope of this study, this perspective raises the possibility that the divergence between HP and KO populations reflects the differential maintenance of maternal sensitivity, consistent with the rarely confirmed theoretical expectations that plasticity is maintained only when its benefits outweigh its costs [11,13,27,29].

Taken together, our findings suggest that the evolution of TGP cannot be understood without considering the functional role of the focal trait within a life-history context. In our system, developmental timing, rather than offspring size, emerged as the primary axis through which maternal effects were expressed. Traits governing when offspring enter the environment appear to be especially sensitive to condition-dependent maternal effects (figure 5), whereas traits governing offspring performance remain largely under within-generational plastic control. This distinction may help reconcile why many empirical studies and meta-analyses detect strong maternal effects but provide ambiguous support for anticipatory matching across generations [12,51,79,80]. Importantly, we show that the strength of maternal effects differs with populations of killifish from ecologically divergent sites. Rather than evolving predictive forecasting, the populations have diverged in how tightly the offspring developmental rate is coupled to maternal condition. We simply reiterate that life-history evolution probably modifies the strength of maternal effects through resource mediation, whereby the maternal energetic state itself serves as a reliable indicator of prevailing ecological conditions. More broadly, our results suggest that condition-dependent maternal effects may represent a primary adaptive pathway in systems in which selection acts strongly on reproductive timing [46]. Recognizing this distinction shifts the focus from asking whether anticipatory effects are adaptive to asking which life-history dimensions are most likely to be shaped across generations.

## Supplementary Material



## Data Availability

The data associated with this study are publicly available through the Dryad Digital Repository [62]. Supplementary material is available online [81].
