# Supplementary material for: Evolutionary shifts in condition-transfer effects but not anticipatory maternal effects in Trinidadian killifish (Anablepsoides hartii)
Source: Proc Biol Sci. 2026 Nov 4;293(2082):20261362. doi: 10.1098/rspb.2026.1362 (PMC13730799; doi:10.1098/rspb.2026.1362)
Supplement: Supplementary file 1 [file rspb.2026.1362.zip › rspb.2026.1362/rspb20261362_si_001.docx]

**Supporting Information for**

Evolutionary shifts in condition-transfer effects but not anticipatory maternal effects in Trinidadian killifish (*Anablepsoides hartii*).

*Proc. Roy. Soc. B. 2026*

Marcus A. Lee^1,2,†^, Amanda J. Stoner^2^, Christopher Roden^2,3^, Matthew R. Walsh^2^

^1^Functional Ecology, Lund University, Sweden

^2^Department of Biology, University of Texas at Arlington, USA

^3^Department of Biology, University of Miami, USA

^†^Corresponding author: Marcus A. Lee,

**Email:**  marcus.lee@biol.lu.se

Figure

Fig. S1. Heatmap denoting the additive genetic relatedness between individuals used in this study. No pairwise relatedness value exceeded 0.5, indicating no inbreeding or duplicate individuals. River-level populations (Arima, AM; Aripo, AP; El Cedro, EC) are delineated by black lines. Within each river, two sub-blocks are visible corresponding to the high-predation (HP) and killifish-only (KO) populations; however, these are not explicitly labelled in the heatmap. The block-diagonal structure reflects the absence of outcrossing among populations.

**Figure S2**. Variation in egg development time among rivers. Small, semi-transparent points show the raw observations for each effective river. Large points represent model-adjusted mean egg development times estimated from the Poisson generalized linear model, with error bars indicating 95% confidence intervals. Estimates account for F2 food level, F3 food level, population, and the interaction between F2 food level and population. Colours distinguish effective rivers.

Tables

Table S1. Summary of family structure within the analysed pedigree. The table shows the number of unique dams, sires, and dam × sire crosses (families) contributing to each river–population combination, together with the number of analysed individuals and the distribution of family sizes. This information is provided to document the pedigree structure and variation in family size represented in the animal models.

| River | Population | F0 Dams | F0 Sires | Families | F3 Individuals | Mean family size | Median family size | Family size range |
| --- | --- | --- | --- | --- | --- | --- | --- | --- |
| AM | HP | 5 | 6 | 7 | 109 | 15.6 | 13 | 1-33 |
|  | KO | 6 | 6 | 8 | 96 | 12 | 11.5 | 2-23 |
| AP | HP | 11 | 9 | 19 | 249 | 13.1 | 8 | 2-43 |
|  | KO | 9 | 9 | 15 | 205 | 13.7 | 12 | 1-39 |
| EC | HP | 9 | 9 | 15 | 176 | 11.7 | 11 | 3-31 |
|  | KO | 5 | 6 | 7 | 83 | 11.9 | 9 | 1-38 |

Table S2. Sample sizes for each treatment combination used in analyses of F3 weight, F3 body condition, and egg development rate across population (high predation, HP; killifish-only, KO), parental food level (F2), and offspring food level (F3). Values indicate the number of individuals included in each model following removal of missing data.

| Population | F2 food level | F3 food level | F3 weight | F3 body condition | Egg development rate |
| --- | --- | --- | --- | --- | --- |
| HP | HF | HF | 201 | 201 | 158 |
|  |  | LF | 169 | 169 | 126 |
|  | LF | HF | 87 | 87 | 69 |
|  |  | LF | 74 | 74 | 65 |
| KO | HF | HF | 153 | 153 | 118 |
|  |  | LF | 133 | 133 | 114 |
|  | LF | HF | 59 | 59 | 46 |
|  |  | LF | 34 | 34 | 21 |

**Table S3**. Comparison of Bayesian model estimates with and without river of origin as a covariate. Due to the similarity, the simpler model was selected for the final analysis in the manuscript.

|  | Weight model | | | Body condition model | | |
| --- | --- | --- | --- | --- | --- | --- |
| Parameter | With River | Without River | Interpretation | With River | Without River | Interpretation |
| Intercept | 3.50 | 3.52 | Essentially identical | -0.13 | -0.13 | Identical |
| F3 Food | -1.11 | -1.11 | Identical | -0.01 | -0.01 | Identical |
| F2 Food | -0.01 | -0.01 | Identical | -0.04 | -0.04 | Identical |
| Population | 0.06 | 0.05 | Negligible change | 0.01 | 0.01 | Identical |
| F3 × F2 | -0.12 | -0.12 | Identical | -0.60 | -0.60 | Identical |
| F3 × Population | -0.13 | -0.13 | Identical | -0.03 | -0.03 | Identical |
| F2 × Population | -0.04 | -0.04 | Identical | 0.03 | 0.03 | Identical |
| Three-way | 0.01 | 0.01 | Identical | -0.06 | -0.06 | Identical |
| σ | 0.40 | 0.40 | Identical | 0.28 | 0.28 | Identical |
